# Sudan’s Mpox surveillance system during the 2022 pandemic: a retrospective evaluation

**DOI:** 10.3389/fpubh.2025.1669389

**Published:** 2025-11-06

**Authors:** Ahmad Izzoddeen, Omer Elbadri, Magam Musa, Alaa Hamed Dafaalla, Mazza Abasher, Mustafa Magbol, Muntasir M. O. Elhasan, Mohamed Nageeb Abdalla

**Affiliations:** 1 Field Epidemiology Training Program (FETP), Federal Ministry of Health (FMOH), Khartoum, Sudan; 2 FMOH, Khartoum, Sudan; 3 Faculty of Medicine, Al-Zaiem Al-Azhari University, Khartoum, Sudan

**Keywords:** Mpox, communicable disease surveillance, surveillance evaluation, Monkeypox, one health, Sudan priority notifiable diseases

## Abstract

**Background:**

Mpox (formerly monkeypox) is a zoonotic disease caused by the Mpox virus. Although the disease is endemic in several Central and West African countries, it has recently emerged in Europe and the United States and was declared a public health event of international concern. This study aimed to evaluate Mpox surveillance in Sudan and provide insights for better epidemic preparedness.

**Methods:**

Mpox surveillance was evaluated based on Centers for Disease Control and Prevention guidelines. The targeted attributes were flexibility, sensitivity, usefulness, representativeness, timeliness, and data quality (data completeness and adequacy of variables). To evaluate the qualitative attributes (flexibility, usefulness, and representativeness), interviews were conducted with key informants, supported by records and reports review. The national surveillance line-list was obtained for evaluation of the quantitative attributes: timeliness, data quality, and sensitivity.

**Results:**

The surveillance system was flexible enough to integrate Mpox within a short time. A technical committee was formulated, and a preparedness and response plan developed. The case definition was adapted and reporting activated through different surveillance types. Surveillance was useful in detecting Mpox, generating epidemiologic indicators, and guiding preparedness and response interventions. The system showed representativeness geographically and through multiple reporting sources. The case definition was broadly sensitive as it detected Mpox cases and other dermatological conditions, with proven detection capacity by different surveillance types. The line-list lacked important data on medical history and exposure. The timeliness of reporting was good; however, the testing capacity was limited.

**Conclusion:**

The surveillance system demonstrated high flexibility in rapidly integrating Mpox, with sensitivity in detecting cases and representative reporting sources. It was useful in detecting Mpox, generating epidemiologic indicators, and informing actions. Improvement in data quality and completeness is required for in-depth analysis. Rapid response teams’ training and sustainable financing for their operations are highly recommended and crucial for timely investigation, quality data, and specimen collection. Expanding molecular-testing capacity to regional laboratories and strengthening specimen-transport networks are critical, together with shifting to a One Health approach.

## Introduction

Mpox (formerly Monkeypox) is a zoonotic disease caused by the Mpox virus (formerly Monkeypox virus), a virus with an uncertain reservoir, though thought to be small mammals like rodents (1, 2). Two clades of the Mpox virus were recognized: clade I (formerly the Central African or the Congo Basin) and clade II, formerly the West African clade. The outbreak reported in 2022 was linked to clade II subtype, which causes a less severe disease compared to clade I (3–5). Mpox resembles smallpox infection in its clinical spectrum, with features starting with early lymphadenopathy, malaise, headache, and fever (5–13 days after exposure), followed later by a deep-seated, vesicular or pustular, well-circumscribed skin rash that progresses over time to form scabs (6). Most of the infected cases recover within a few weeks; nevertheless, complications are possible, especially among patients with weakened immune systems, children, and pregnant women (7). Although the disease is endemic in Central and West African countries, it has recently emerged in Europe in mid-2022 with a marked increase in cases, reaching over 70,000 by October 2022 (8–10). That has led the World Health Organization (WHO) to declare Mpox as a Public Health Event of International Concern (PHEOIC) in July 2022 (6). In Sudan, the disease was not part of the priority-notifiable diseases’ list; therefore, it was not considered in the surveillance system. Based on the International Health Regulations (IHR), countries have to build strong communicable disease surveillance systems and strengthen their detection capacity, particularly for diseases of international public health concern (11). Sudan’s public-health surveillance system operates within a complex epidemiologic environment marked by frequent zoonotic, vector-borne and other disease outbreaks. The country shares extensive borders with endemic nations such as the Central African Republic, South Sudan and Chad which shares borders with Democratic Republic of Congo, where heightens the risk of cross-border transmission of infectious diseases including Mpoxd. Sudan has already a well-established Indicator-Based Surveillance (IBS) and Event-Based Surveillance (EBS) with a list of 25 priority-notifiable diseases (top priority among them are: cholera, Acute Flaccid Paralysis (AFP), yellow fever, haemorrhagic fevers, neonatal tetanus, plague, Severe Acute Respiratory Syndrome (SARS), epidemic influenza, measles, meningitis, and Guinea worm), and 12 priority public health signals (related to the priority diseases). These diseases were selected based on specific criteria to align with the country specific profile, the most important among these criteria are; disease burden in Sudan, past outbreaks, severity, transmissibility, capacity to detect and capacity to control. Mpox was not part of the top priority with no recent circulation. Additionally, there is an active Early Warning Alert and Response System (EWARS) reporting from the conflict and internal displacement areas. Reporting is also active at the points of entry (POEs). The IBS covers around 2,215 reporting sites distributed in all localities (districts) in the country. The EBS main source of data is villages under community-based surveillance (CBS), which is estimated to be over 5,000 villages. Additionally, EBS reports are also based on media scanning and partner (other ministries and sector partners) notifications. For all systems, the data flows from the lower level (health facility, community) through the locality (district), to the state, and finally to the national level, where the final validation and analysis are done; however, initial analysis and reports are made at the subnational (locality and state levels). Situation reports are developed and disseminated to relevant stakeholders. Integrating a new disease into the surveillance system is challenged by many factors including staff shortage, lack of training, supportive equipment and tools, difficulties in communication, and poor infrastructure (12). This evaluation shares insights from the Mpox surveillance system in Sudan and aims to evaluate the system attributes to inform evidence-based future improvements. Mpox surveillance was established with the objective of ensuring early detection of Mpox cases and providing timely information to guide public health preparedness and response actions.

### Methodology

*Study design*: This was a cross-sectional, institution-based study (both qualitative and quantitative).

*Study area and population*: The study was carried out in Sudan at the Federal Ministry of Health (FMOH) and targeted the national surveillance case records of Mpox in the national line list. It also included eight key informants: the national surveillance director, two surveillance officers working in Indicator-Based Surveillance (IBS), two Event-Based Surveillance (EBS) focal points, two Mpox surveillance national focal points, and the emergency response director. These individuals were involved in Mpox surveillance and control at the Health Emergencies and Epidemic Control Directorate (HEEC).

*Data collection and variables*: The Mpox case definition adopted by the FMOH defines a suspected case as a person of any age presenting with an unexplained acute rash and one or more of the following signs or symptoms: headache, acute onset of fever (>38.5 °C), myalgia, back pain, asthenia, or lymphadenopathy. A confirmed case is one that meets the case definition and is laboratory-confirmed for Mpox virus by detecting unique sequences of viral DNA through Real-time Polymerase Chain Reaction (PCR) or sequencing. For tested cases, only RT-PCR was used for confirmation.

Due to the unavailability of Mpox testing in Sudan, outbreak confirmation relied on testing initial samples (25 samples with 18 positive RT-PCR results for MPXV) outside the country. Subsequent cases continued to be reported based on the standard case definition and clinical diagnosis.

The study followed the guidelines of the Centers for Disease Control and Prevention (CDC) for surveillance system evaluation. Both qualitative and quantitative attributes were assessed. For the qualitative part, the evaluation included in-depth interviews with key informants based on CDC guidelines and a review of surveillance documents and reports: (I) Flexibility: The ability and responsiveness of the system to include Mpox and the adaptive actions taken, such as adopting the case definition, developing reporting forms, and organizing plans and training for staff. (II) Usefulness: The system’s capacity to detect Mpox cases, generate epidemiologic indicators, and inform and guide actions based on system data and reports. (III) Representativeness: Evaluated by the representativeness of reporting sources and sites across different geographical areas in the country and the involvement of complementary surveillance types.

For the evaluation of the quantitative attributes, the Mpox national surveillance line list was obtained, and the targeted data were extracted through an extraction form. The extracted variables included case ID, state and locality, date of symptoms’ onset, date of reporting, date of specimen collection, date of testing, lab result, and the result date. The epidemiologic reports were reviewed for the inclusion of surveillance indicators and types of the system that contributed to detection of cases. This evaluation focused mainly on sensitivity and timeliness. Other line-list variables were compared with the standard recommended variables for data collection, as recommended by the CDC and WHO, to evaluate the adequacy of variables and to check for their missing values to evaluate completeness.

*Sensitivity*: evaluated quantitatively through assessing the sensitivity of the case definition to detect Mpox cases (measured by comparing the number of true cases to other cases detected by the system) and the sensitivity of the different surveillance types to detect the disease (the proportion of cases detected by each type).

*Timeliness* was assessed in terms of: firstly, the time between detection and reporting (within 24 h was considered on-time, 24–48 h was considered late, and more than 48 h was considered very late); secondly, the time between reporting and specimen collection (same as reporting); and finally the time from collection to test result (test result within 5 days was considered on-time, 5–7 days was considered late, and more than 7 days was considered very late).

*Adequacy of variables* was assessed by comparing the data collected to the data recommended by the WHO and CDC. Completeness of data was assessed by checking the number of missing values for each variable in the national line list.

*Data analysis*: quantitative data were customized through Microsoft Excel software and then imported into Epi Info 7 software for analysis. Frequencies and percentages were used, and data were presented in tables. Ethical approval and permission were obtained from the Health Emergency and Epidemic Control Directorate (HEEC) at the Federal Ministry of Health (FMOH).

*Ethical considerations*: this work was done as an assignment under the Field Epidemiology Training Program (FETP), for which the approval to conduct the study was granted from the HEEC General Directorate. The data used were secondary data provided in anonymized form and after removal of all personal information. The interviewed informants were staff already working under the same directorate. Other attributes, such as simplicity and acceptability, though important for providing additional clarity and deeper understanding, were not included in this evaluation due to data limitations and contextual constraints.

## Results

On May 22, 2022, the first suspected case was reported from West Darfur State, specifically from the Forbaranga locality. This is a border locality that shares borders with Chad and the Central African Republic. Up to that date, Sudan had no surveillance system in place for Mpox.

Being aware of the emergence of the disease in Europe, the HEEC Directorate started to establish a surveillance system for the disease, even 1 month before its declaration by the WHO. The outbreak extended for 24 weeks and ended in October 2022.

There were 375 reported cases from 17 out of 18 states, with the exception of the Northern State. The highest number of cases was reported from Gadaref (45.3%), West Darfur (25.9%), Khartoum (13.3%), and North Darfur (3.5%).

### Flexibility (adaptability)

In late May, the reported suspected Mpox case prompted the health authorities to prepare for a potential spread of the disease. In the same month, a technical committee was formulated at the national level and named the National Mpox Preparedness and Response Committee. Following this, a national Mpox preparedness and response plan was developed, addressing several pillars: surveillance, preparedness, and response.

The committee developed and adapted a case definition based on CDC and WHO definitions. A factsheet was then developed, which included the case definition, reporting procedures, management guidelines, and preventive measures, and this was distributed to states and localities. Additionally, a case investigation form (Case Report Form - CRF) was developed and disseminated to facilitate case investigation and data collection.

Zero reporting (reporting the disease as zero cases, even when no case was detected) from all sentinel sites under IBS was activated. Community-based surveillance (CBS) was also included to enhance detection capacity, with volunteers oriented and encouraged to report any relevant signals.

A focal point specifically for Mpox surveillance was assigned at the national level to follow up with states, record and analyze data, and write reports. Laboratory testing capacity was limited during the first 2 months. This was managed by coordinating with external laboratories, and specimens were sent and tested abroad. This issue was resolved 2 months later when testing reagents became available, and specimens were tested in the National Public Health Lab (NPHL).

In August 2022, case reporting formats were printed and distributed to all states. All the above-mentioned adaptive steps and activities reflect and demonstrate the flexibility of the surveillance system to include the emerging Mpox. No challenges regarding the data flow and reporting pathway were identified or mentioned. The explanation for that is possibly because Mpox surveillance utilized the already existing data flow and reporting pathway of other diseases to which the staff at the different level are familiar and adapted.

### Usefulness

**Figure 1:**
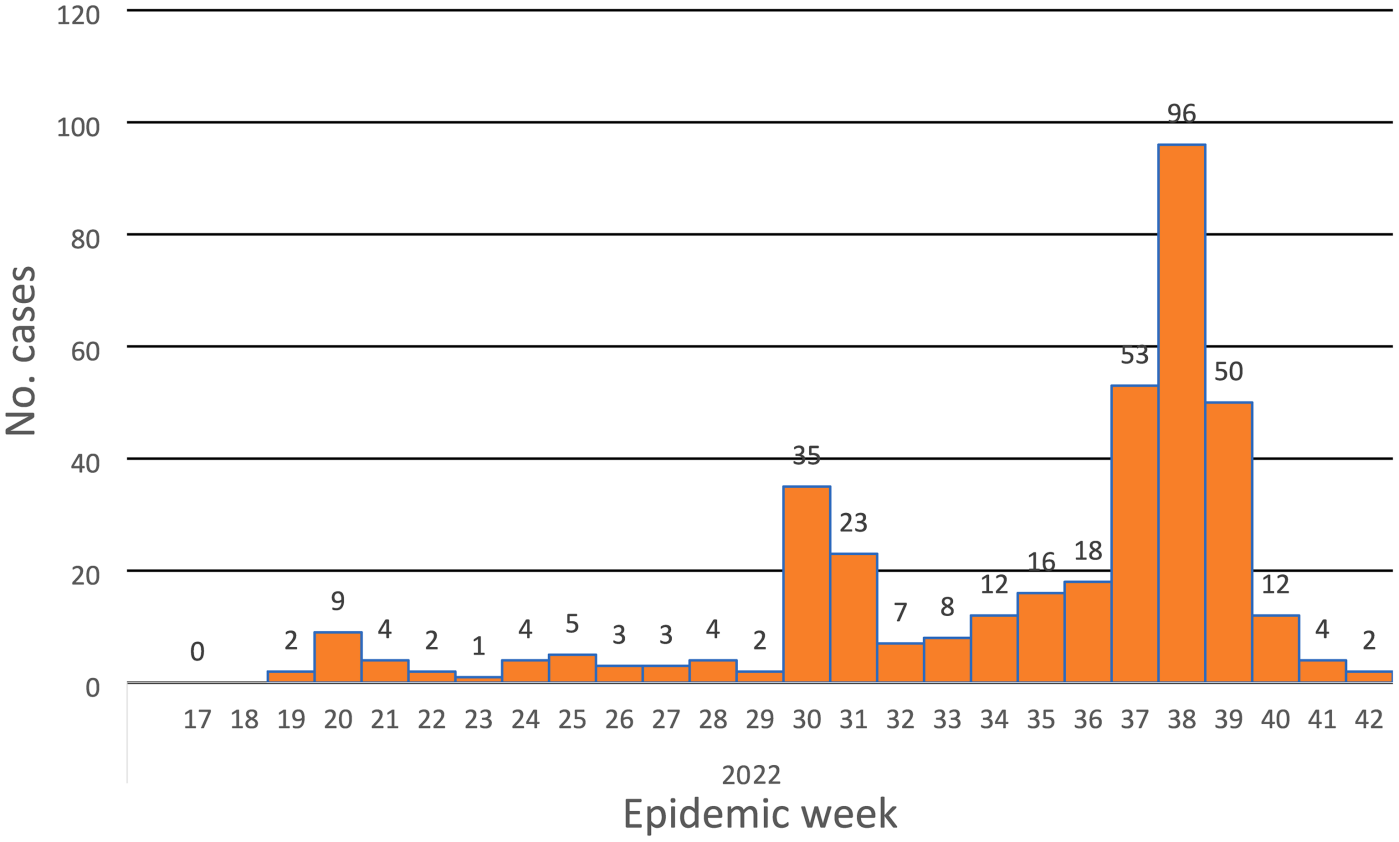
The epidemic curve of Mpox epidemic in Sudan, 2022 (*n* = 375).

**Figure 2:**
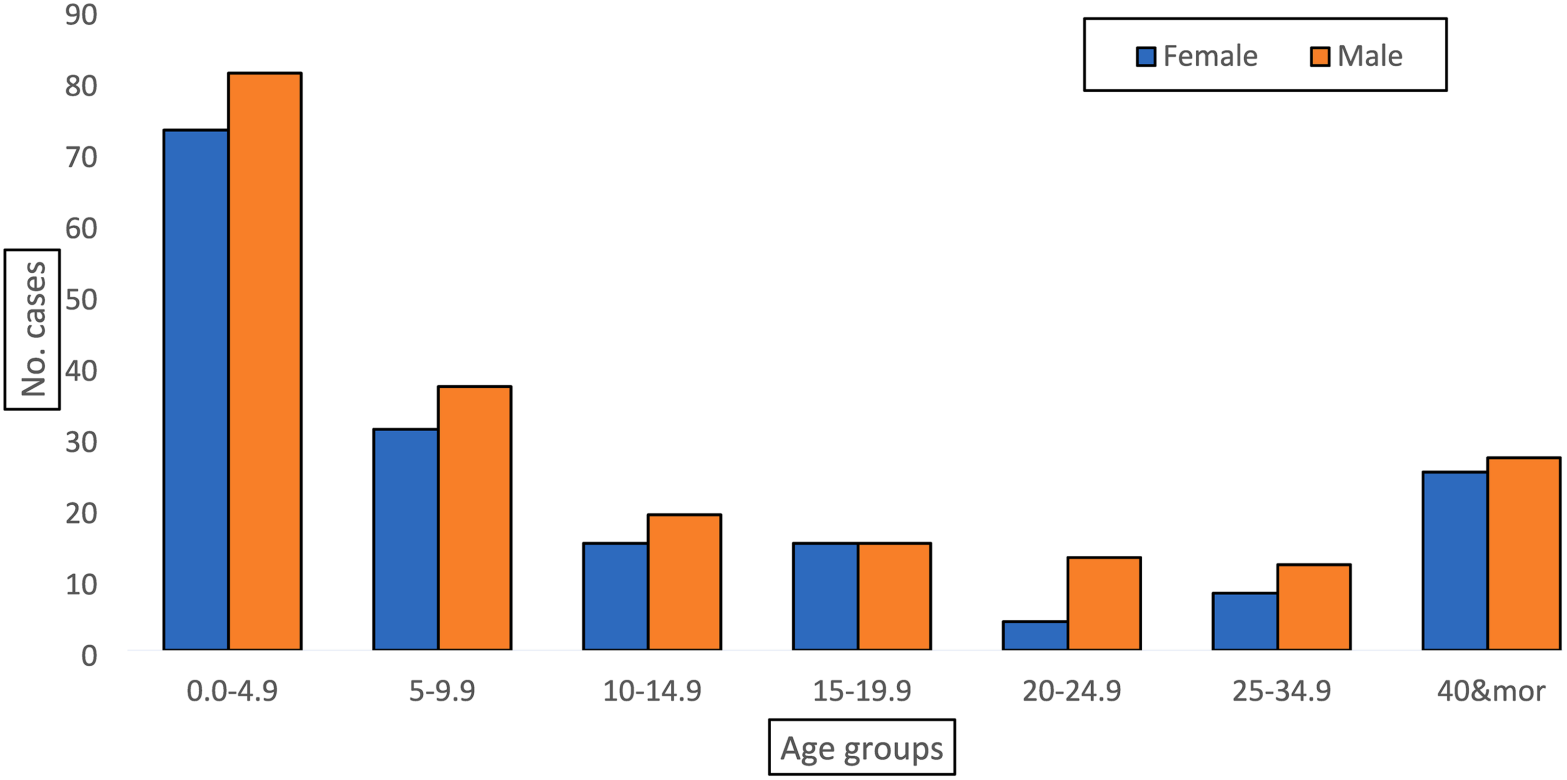
The distribution of Mpox cases by age group and gender, Sudan, 2022 (*n* = 375).

The system revealed its usefulness in detecting the disease and providing valuable epidemiologic indicators to inform decisions. From the line-list, we calculated the overall attack rate (2.36 cases/100,000 population at risk), the positivity rate for the tested specimens (72% positivity rate), time trends of the disease (Figure 1), place distribution, and age distribution (Figure 2). All of these indicators were well represented in the epidemiologic reports from the surveillance directorate and shared with the relevant stakeholders.

Interviews with surveillance staff also revealed the usefulness of the system. This was reflected by the number of decisions made based on the weekly or daily reports presented to the Emergency Operation Center (EOC), which is headed by the federal health minister. Five rapid response teams (RRTs) were deployed in the affected areas to investigate cases and apply control measures. In addition, budget allocation was made for affected states to facilitate the response, and finally, three supportive supervision missions were conducted. All of these actions were based on the surveillance data.

Moreover, reports were also presented to the Mpox committee on a regular basis. Training for point-of-entry personnel was implemented to capture imported cases entering the country through the different portals. Reports from the national focal point were used regularly to improve the functionality of the system.

### Representativeness

Mpox cases were reported from all the sentinel sites under IBS. These selected health facilities represent 32% (2,215/6,957) of all health facilities in Sudan and were selected primarily based on standardized criteria to ensure representation across all states and localities in the country.

Furthermore, zero reporting (meaning regular reporting even if no cases were detected) was required by all sites on a daily basis. Supporting this, there was a functioning CBS in 16 out of 18 states. This system covered areas without health facilities or where facilities were not part of IBS, making it complementary to IBS. Volunteers under CBS reported dermatological syndromes (any person/s with fever, skin lesions with/without itching).

Lastly, Sudan adopted and implemented EWARS, which monitors the epidemic diseases situation among risk groups like Internally Displaced Persons (IDPs), refugee communities, and conflict-affected areas. EWARS contributed to the detection of 25% of reported cases from refugee camps in Gedaref State.

### Sensitivity

The case definition was highly sensitive and captured various cases of other dermatological diseases. Out of 25 specimens tested, 18 were positive (72% positivity rate). From the 375 reported suspected cases, 30% were diagnosed as scabies, 10% as chickenpox, and 4% as other dermatological problems. Sensitivity was also assessed based on the proportion of cases detected by the different types of surveillance, as this reflects the sensitivity of the different systems in detecting Mpox. The reviewed surveillance reports showed that 65% of cases were detected at IBS sentinel sites, 7% by community volunteers through CBS, 25% from displacement and refugee camps via EWARS, and 3% at points of entry.

### Completeness

*Quality of data (adequacy/representativeness of variables)*: The data collected for Mpox suspected cases lacked important variables that are very important for the analysis. These important variables include medical history, exposure, and others listed in Table 1.

**Table 1:** Demonstrates the missing variables in the national Mpox surveillance line-list.

Demographic
Marital status
Education
Workplace
Medical Hist.
Pregnancy status (for female)
Comorbidities
Chronic medications
Anogenital rash
Oral/mucus
Description of rash
Other symptoms
Hospitalization
ICU admission
Complications
Exposure
Contact settings
Detailed exposure
Traveling history/ destination
Contact with animals/types of animals
Specimen
Type of specimen collected
Type of test

### Completeness of data

Regarding the completeness of the data entered, 9 variables in the line-list were identified to have missing values (Table 2). About 36.1% of cases had missing occupation data. About 75.7% percent of cases had no contact numbers for follow-up and communication if further data were needed.

**Table 2:** Represent the frequency of missing entries in line-list variables.

Variable	Missing frequency (%)
Occupation	125 (36.1%)
Date of onset	5 (1.4%)
Date of result	2 (0.58%)
Date of specimen	6 (1.7)
Label number	3 (0.87%)
Sample not taken	163 (45%)
Specimen not sent	10(3%)
No phones	262 (75.7%)
Residence	3 (0.87%)

Timeliness was assessed in terms of the time from detection to reporting, reporting to specimen collection, and from collection to the appearance of the test result (Table 3). Reporting: Out of the 375 suspected cases, 61% of cases were reported on time (within 24 h.), 14% were reported late (24 h–48 h.), and 24% were reported very late (more than 48 h.). Specimen collection: Specimens were collected from only 212 of the suspected cases. 94.8% of the specimen collection occurred in a reasonable time (24–48 h.). Twenty-five (11.7%) of the specimens were tested for Mpox virus using RT-PCR by the NPHL. The number of specimens tested was small as the lab capacity was limited at the time of the study where the NPHL depended on another lab outside Sudan. Later the lab capacity improved. The time taken to identify the virus was more than 7 days, which classified testing as very late.

**Table 3:** Timeliness of surveillance in terms of, reporting, sampling and specimen testing.

Timeliness		Frequency	Percent (%)
Reporting	On time (up to 24 h)	231	61.6
	Late (> 24–48 h)	53	14.1
	very late (> 48 h)	91	24.3
		375	100
Specimen collection	On time (up to 24 hours)	201	94.8
	Late (> 24–48 h)	1	0.5
	very late (> 48 h)	10	4.7
		212	100
Result	On time (< 5 days)	–	–
	Late (5–7 days)	–	–
	very late (> 7 days)	25	100
		202	100

## Discussion

Very shortly after the striking appearance of Mpox in Europe, Sudan’s surveillance system began to integrate the disease. Efforts by the HEEC started by formulating the Mpox Preparedness and Response Committee at the national level. That is quite reflective of the system’s responsiveness and flexibility to consider the disease in the priority-notifiable diseases list. Generally, following the declaration of pandemics, taking COVID-19 as an example, many countries immediately integrated COVID-19 into their surveillance using different approaches (13). When Mpox was declared by the WHO as a PHEIC, there was an already functioning surveillance system for Mpox in Sudan, a focal person assigned, and a daily zero reporting from health facilities (IBS), and EBS represented by community (CBS), EWARS sites, and POEs. These are the same surveillance types adopted by several African countries for other pandemic diseases (14). In addition, CBS proved its effectiveness outside Africa as well (15). An example from Cameroon showcases the importance of relying on both clinicians and community workers to enhance the detection capacity (16).

Generally, collaborative surveillance and multi-sector coordination are key and highly recommended as strategic approaches to best tackle the Mpox risk (17). The new advancements in the field require strengthening the One Health platform to ensure multi-sectoral collaboration and coordinated effort in Sudan. The system is useful in detecting Mpox (18) and informing actions as it provides estimates and epidemiologic indicators about the disease, and reports were timely shared with relevant directorates and partners. In addition to the ability to detect the target disease, it is well known that surveillance systems’ usefulness is also measured by the actions taken and decisions made based on the reported data (19).

The efforts by the HEEC directorate were to activate reporting from the different functioning surveillance types to ensure system representativeness. This was done with zero reports received from all sentinel sites, representing 32% of the health facilities all over Sudan. This is aided by daily reports from community volunteers in the CBS, and reports from POE and EWARS. Based on that, the surveillance of Mpox is not different from any other notifiable disease in the country, and the system is representative. Representativeness of the data and coverage by all sources is generally ensured by adopting different surveillance types to ensure complementarity. This was reported in many countries when they experienced pandemics (14, 20).

The MoH developed a highly sensitive case definition to not miss any case, and this sensitivity is reflected by the positivity rate (72%) and the other reported dermatological diseases like scabies, chickenpox, and measles. The surveillance system in epidemic settings is better designed to be based on broad definitions to increase the detection capacity and to not miss any single case (21), this was also reported from Cameroon (16). Our study findings are, as well, comparable to those reported in the Democratic Republic of Congo, where an 80% sensitivity was observed in detecting cases based on patients’ symptoms and demographics, along with other dermatological diseases such as chickenpox (22).

It is very important for the surveillance system to collect data on medical history, especially comorbid conditions, chronic medicines, and exposure, as they are well known to increase the risk of contracting infections. This will help in identifying the high-risk groups and guide the control efforts to eliminate the exposure. The data and the analysis reports lacked these important variables. In addition, there was also a high percentage of missing values in the occupation variable (75.7% missing). Completeness of data records is key to achieving better analysis. Generally, the reporting timeliness needs improvement as 38.45% of cases were reported late or very late. This can be achieved through supportive supervision to reporting sites to identify the actual factors behind the delay, and can also be addressed by the provision of communication costs. Timeliness in Mpox surveillance remains a challenge as highlighted by Delia et al. in Cameroon, as well as data quality (16). The time from reporting to specimen collection is generally reasonable, however, it could be further improved by the provision of good training to the rapid response teams (RRT), and maintaining regular coverage of the operational costs for their timely deployment. According to the testing strategy, confirmation of Mpox was done through RT-PCR at the NPHL in Khartoum, the central reference lab for diagnostics. While the NPHL had technical expertise and trained personnel, its capacity was constrained by limited reagents, restricted cold-chain transport, and the absence of regional testing hubs. These factors resulted in delayed testing and confirmation. Expanding molecular-testing capacity to regional laboratories and strengthening specimen-transport networks would substantially improve diagnostic timeliness and data completeness for Mpox and other emerging zoonoses. Defective timeliness in surveillance is a recognized area of weakness mainly in low-income countries due to the lack of resources and training (13). The establishment of regional laboratories or zonal ones in other states to facilitate testing and to decrease the logistical cost is a proposed strategy.

## Conclusion

The surveillance system was flexible to include the emerging Mpox in a reasonable time with sensitivity in detecting cases, and representative reporting sites and sources. The usefulness was evident through the cases detected, epidemiological indicators generated, and the actions taken based on the system outputs. Improvement in the data quality and completeness is required for in-depth analysis to inform actions. Rapid response teams’ training and maintenance of their operations financing are highly recommended and crucial for timely investigation, quality data, and timely specimen collection. Expanding molecular-testing capacity to regional laboratories and strengthening specimen-transport networks would substantially improve diagnostic timeliness and data completeness for Mpox and other emerging zoonoses. Strengthening the One Health platform is critical to ensure the multisectoral collaboration and coordinated effort in Sudan.

### Study limitation

The lack of detailed data from the laboratory stands against more detailed analysis on the sensitivity, regarding predictive value (positive and negative). Moreover, limited access to data on response actions and mitigation activities restricted the provision of better insight into the epidemic response in the country.

## Data Availability

The data analyzed in this study is subject to the following licenses/restrictions: the data is available in data archive of federal ministry of health, Republic of Sudan, and accessible by researchers upon request. Requests to access these datasets should be directed to muntasiro@yahoo.com.
